# Simple method for cutoff point identification in descriptive high-throughput biological studies

**DOI:** 10.1186/s12864-022-08427-6

**Published:** 2022-03-14

**Authors:** Alexander Suvorov

**Affiliations:** Department of Environmental Health Sciences, School of Public Health and Health Sciences, University of Massachusetts, 686 North Pleasant Street Amherst, Amherst, MA 01003 USA

**Keywords:** Cutoff, Dichotomization, Descriptive genomics, Threshold, -omics

## Abstract

**Background:**

Rapid development of high-throughput omics technologies generates an increasing interest in algorithms for cutoff point identification. Existing cutoff methods and tools identify cutoff points based on an association of continuous variables with another variable, such as phenotype, disease state, or treatment group. These approaches are not applicable for descriptive studies in which continuous variables are reported without known association with any biologically meaningful variables.

**Results:**

The most common shape of the ranked distribution of continuous variables in high-throughput descriptive studies corresponds to a biphasic curve, where the first phase includes a big number of variables with values slowly growing with rank and the second phase includes a smaller number of variables rapidly growing with rank. This study describes an easy algorithm to identify the boundary between these phases to be used as a cutoff point.

**Discussion:**

The major assumption of that approach is that a small number of variables with high values dominate the biological system and determine its major processes and functions. This approach was tested on three different datasets: human genes and their expression values in the human cerebral cortex, mammalian genes and their values of sensitivity to chemical exposures, and human proteins and their expression values in the human heart. In every case, the described cutoff identification method produced shortlists of variables (genes, proteins) highly relevant for dominant functions/pathways of the analyzed biological systems.

**Conclusions:**

The described method for cutoff identification may be used to prioritize variables in descriptive omics studies for a focused functional analysis, in situations where other methods of dichotomization of data are inaccessible.

**Supplementary Information:**

The online version contains supplementary material available at 10.1186/s12864-022-08427-6.

## Introduction

Descriptive omics represent one particular type of study in which a big number of continuous biological variables (e.g. genes, proteins, metabolites) are measured in a biological sample to characterize it rather than to compare it with other samples (e.g. treatment groups, disease states). Descriptive studies provide background knowledge for future research as they characterize biological systems at molecular levels. As such, descriptive omics is analogous to the effort of XVIII century biologists in building a descriptive fundament for organismal-level biology. Today descriptive omics results in many essential resources of medico-biological research such as databases providing quantitative information on genes, proteins, sncRNA, metabolites, and other biological variables across many organisms, tissues, cell types, and biological liquids. Extraction of biologically meaningful information from these resources may be challenging.

One approach is based on an assumption that a small number of variables with the highest values of expression/abundance dominate functions of a biological system. For example, it is reasonable to assume, that genes with high expression values are more important for the normal tissue physiology than these expressions of which is close to zero. This approach requires methods of cut-off point identification to generate shortlists of variables for focused analysis.

Several methods of dichotomization were developed previously by different research domains as a result of the rapid development of high-throughput omics and other technologies and approaches in the medico-biological domain. For example, a big group of existing methods identifies cutoff points based on an association of continuous variables with other biologically meaningful variables. For example, a widely used approach for the identification of genes differentially expressed in relation to a health condition or treatment utilizes fold-change and false-discovery rate adjusted *p*-value as cutoff criteria. A range of algorithms and online tools was developed to categorize variables for decision-making about cancer treatments [1–3]. These approaches do not apply to descriptive studies in which associations with other biologically meaningful variables are not known.

Another group of methods was developed for image segmentation. For example, Otsu’s method was developed to separate pixels in an image into two classes: object and background [4]. Global thresholding algorithms for image segmentation, including Otsu’s, perform well only when the distribution of continuous variables is close to bimodal (comparable number of pixels for object and background and a deep valley between them) [5, 6]. The most common distribution of continuous variables in omics descriptive studies is very different from bimodal, with a majority of variables having very low levels and a very small number of variables having very high values of expression/abundance (see example in Supplemental Fig. 1).

**Fig. 1:**
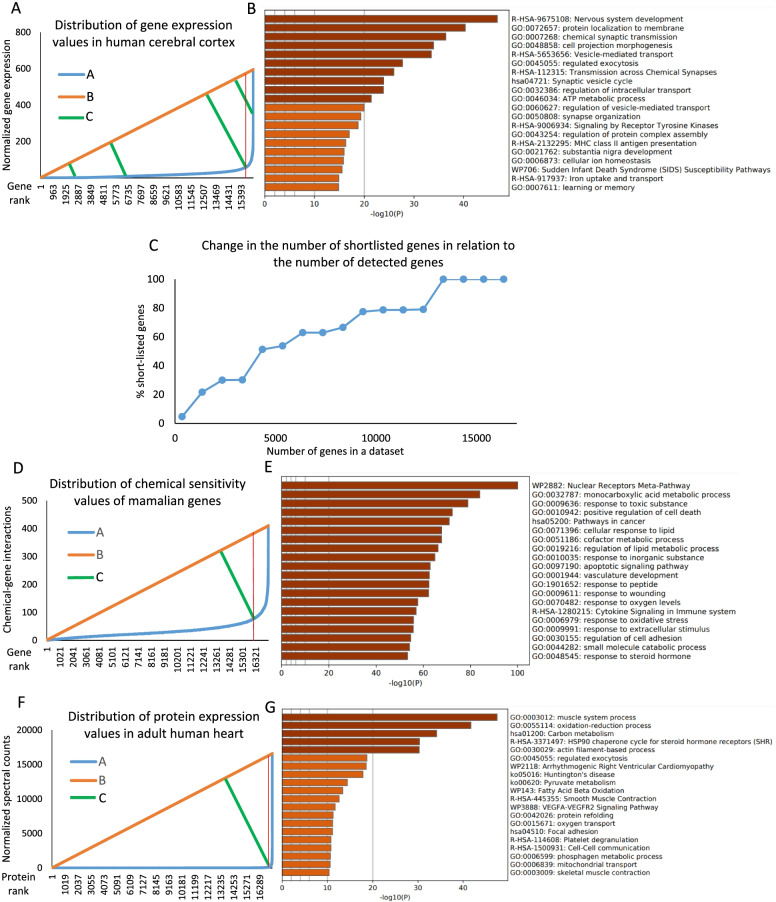
Illustration of the method for cutoff point identification in descriptive high-throughput biological studies. Variable distribution (**A**, **D**, **F**) and biological categories enriched in shortlists identified using cutoff points (**B**, **E**, **G**) for the following datasets: genes expressed in the human cerebral cortex (**A**, **B**), genes sensitive to chemical exposures (**D**, **E**), and proteins expressed in the adult human heart (**F**, **G**). Figure C illustrates changes in the number of shortlisted genes identified by the described cutoff algorithm in relation to the number of genes in the dataset. Number of shortlisted genes is shown as percent of the total shortlisted genes identified for a complete dataset (16,353 genes). In graphs (**A**, **D**, **F**), *A* is a curve of the original data distribution, *B* is a linear shortcut connecting the first and the last points of *A*, and *C* is a family of linear functions perpendicular to *B*. Four *C* functions are shown in figure A. In figures C and D longest segments corresponding *C* functions are shown. Red vertical lines in figures A, D, F correspond to the cutoff points

Several methods of dichotomization of droplets with and without cell RNA based on the content of their unique molecular identifier (UMI) were developed in a framework of single-cell sequencing technology. Although the distribution of UMI in droplets is continuous, methods used for dichotomization are based on the presence of 2 classes of droplets (empty and non-empty) allowing for the calculation of thresholds based on the deviation from UMI prediction for one class or another [7, 8]. Although methods of dichotomization of continuous variables have broad use in different research domains, Thus, I was not able to identify any method that can be easily applied to descriptive omics data.

The most common shape of the ranked distribution of continuous variables in high-throughput descriptive studies corresponds to a biphasic curve (Fig. 1A, D, F), where in a first phase a big number of variables have low values. These values increase slowly with the rank. In the second phase, a relatively small number of variables demonstrate very rapid growth with their rank number. Thus, the curve of this distribution has a bending point, which delineates the boundary between phases. This boundary may be used as a cutoff point to identify these variables, which dominate the dataset. However, these curves are described by complicated functions, making identification of the inflection point a challenging task. The complexity of that task is the likely reason why I was not able to identify any study using curve fitting to identify cutoff points in descriptive –omics. Here I present a simple method for the identification of the bending point of the curve. This algorithm may be used to identify in an unbiased way variables (genes, proteins, metabolites, etc.) that dominate biological system in a descriptive omics dataset.

## Method description

First, the expression/abundance of all values in the ranked dataset are plotted to generate curve *A*. If we connect the first and the last points of the typical biphasic distribution curve (*A*) of a descriptive omics dataset by a straight line (*B*), together these 2 curves will produce a figure resembling a triangle (Fig. 1A, D, F). Then, for every *x*_*A*_ value of the *A* curve we can calculate a length of a segment that will be perpendicular to the short-cut function (*C*) (Fig. 1A). The longest segment will cross the *A* distribution curve in its bending point.

The B function is a linear function: *y*_*B*_ = *m*_*B*_*x*_*B*_ + *b*_*B*_. Functions perpendicular to *B*, all have the following generic equation: *y*_*C*_ = *(-1/m*_*B*_*)x*_*C*_ + *b*_*C*_. Given the coordinates of crossing points between *A* curve and every *C* function are known (*x*_*AC*_ = rank number of the variable, *y*_*AC*_ = value of the variable (expression, concentration, abundance, etc.)), *b*_*C*_ can be calculated for each such crossing point:

1 $${b}_{C} = {y}_{AC} - (-1/{m}_{B}) {x}_{AC}$$

Thus, now for every point of the *A* curve we have an equation of a linear function *C* that is crossing *A* in that point and is perpendicular to the short-cut line *B*. Now we need to identify coordinates of points at which *B* and *C* functions intersect. Given that coordinates of both functions are the same at intersection, we can equate *x* for both functions: *(y*_*CB*_* – b*_*B*_*)/m*_*B*_ = *(y*_*CB*_* – b*_*C*_*)/(-1/m*_*B*_*)*. From that equation, we can calculate *y* for intersection:

2 $${y}_{CB} = ({b}_{B} + {b}_{C}{{m}_{B}}^{2})/(1 + {{m}_{B}}^{2})$$

As we know *y* for intersection, we can calculate *x* for intersection as well, using an equation for *B*:

3 $${x}_{CB}=\left({y}_{CB}-{b}_{B}\right)/{m}_{B}$$

Now, as we have coordinates for points of the intersection of each *C* function with *A* (*x*_*AC*_*,y*_*AC*_) and coordinates for intersection of each *C* function with *B* (*x*_*CB*_*,y*_*CB*_) we can calculate the length of segments using the Pythagorean theorem:

4 $$D=\sqrt{{({x}_{CB}- {x}_{AC})}^{2}+{({y}_{CB}- {y}_{AC})}^{2}}$$

Given that *x*_*AC*_ is a rank number of the variable, and *y*_*AC*_ is a value of the variable let’s substitute *x*_*AC*_ with *R*, and *y*_*AC*_ with *V.* Let’s also insert Eqs. 1, 2, and 3 into Eq. 4. After simplification, we get the following final equation for the calculation of the length of *D* segments:

5 $$D= \sqrt[2]{\frac{{(V{m}_{B}-{b}_{B}{m}_{B}-R{m}_{B}^{2})}^{2}+ {({b}_{B}+ {m}_{B}R-V)}^{2}}{{(1+ {m}_{B}^{2})}^{2}}}$$

The longest segment will cross *A* curve in the point of the curve bending.

### Examples of the method use

#### Example 1: Identification of genes highly expressed in the human cerebral cortex

Data on consensus normalized gene expression values in the human cerebral cortex were downloaded from The Human Protein Atlas [9]. These values represent the maximum normalized expression values for each gene in three data sources: The Human Protein Atlas, The Genotype-Tissue Expression (GTEx) project [10], and FANTOM5 [11]. The whole dataset consisting of 16,353 genes and their expression values was used in this example. The distribution of expression values ranked from smallest to largest is shown in Fig. 1, curve *A*. The linear function *B* connecting the first and the last points of the curve A has the following equation: *y* = *0.0364x – 0.0364*. Thus, *m*_*B*_ = *0.0364* and *b*_*B*_ = *-0.0364*. These values as well as rank values for every gene (*R*) and normalized expression values for every gene (*V*) were used in Eq. 5 to calculate the length of segments *D* for every gene. The longest segment corresponds to the gene ranked 15,778. This ranking number corresponds to the cutoff point that delineates genes with low and high expression in the human cerebral cortex. To test if highly expressed genes reflect the essential physiology of the cerebral cortex, I submitted the list of top 575 genes determined by the method and ranking 15,779 through 16,353 to Metascape [12] and conducted enrichment analysis with default settings. The enriched biological categories were highly relevant for the nervous tissue physiology and included for example “nervous system development”, “chemical synaptic transmission”, “cell projection morphogenesis”, “cellular ion homeostasis”, and “learning and memory” among others (Fig. 1B). These categories were enriched with a very high level of significance (-log10(*p*) > 15). To control if any genes expressed in the human cerebral cortex are enriched for essential functions of the cerebral cortex, I also submitted to Metascape an equivalent size list of genes with non-zero expression values and lowest expression ranks. This list was enriched for categories non-relevant to brain and nerve tissue, such as “formation of cornified envelop”, “response to bacterium” and “digestion” for example (Supplemental Fig. 2A).

High-throughput methods used today to generate descriptive –omics data often produce datasets of variable size due to the poor detection of low abundance variables. For example, in RNA-seq, genes with very low expression values contribute a small number of reads and may be detected or not by chance. To explore, if this type of variability may have a significant effect on a cutoff point identification I conducted a simulation in which the cutoff point was identified for the whole dataset of cerebral cortex genes and then for reduced lists in which genes were stepwise removed in increments of 1000 starting from the genes with lowest levels of expression (Fig. 1C). This analysis demonstrated that the removal of up to 25% of genes with the lowest expression levels does not affect the cutoff point.

#### Example 2: Identification of genes highly sensitive to chemical exposures

In a recent study sensitivities of genes common to humans, rats and mice were identified based on an overlap of transcriptomic datasets from 2,169 toxicological studies [13]. I use the data from this study available through Mendeley Data [14]. The whole dataset includes 17,338 genes and their respected chemical sensitivity values were used in this example. Chemical sensitivity values here correspond to the number of individual studies with 1,239 chemical compounds in which gene expression was affected by exposure. Following the same steps as in the previous example, I identified the rank number 15,966 as a cutoff point (Fig. 1D). To test if genes sensitive to chemical exposures are associated with known pathways of response to toxicity, I submitted the list of top 1,373 genes determined by the method and ranked 15,967 through 17,338 to Metascape. Identified enriched biological categories included many well-recognized pathways of response to chemical exposures, stress, and damage, for example: “nuclear receptors meta-pathway”, “response to toxic substance”, “apoptotic signaling pathway”, “response to wounding”, “response to oxygen levels”, and “response to oxidative stress” (Fig. 1E). Thus, the cutoff used in this example captured essential molecular mechanisms involved in the response to chemical exposures. These categories were enriched with a very high level of significance (-log10(*p*) > 50). Similarly to Example 1, I looked at the enrichment of the equivalent-size list of lowest-ranking genes. Enriched categories were non-relevant to known mechanisms activated in response to chemical exposures (Supplemental Fig. 2B).

#### Example 3: Identification of proteins highly expressed in the adult human heart

Data on protein expression (at gene level) in the adult human heart were downloaded from The Human Proteome Map portal [15]. These data are based on LC–MS/MS utilizing high resolution and high accuracy Fourier transform mass spectrometry. All mass spectrometry data including precursors and HCD-derived fragments were acquired on the Orbitrap mass analyzers in the high-high mode. The whole dataset including 17,294 unique gene names and expression values of corresponding proteins was used in this example. Expression values were calculated as follows: spectral counts per gene per experiment were first summed from all peptides mapped to each gene. Total acquired tandem mass spectra were used to normalize between experiments and then spectral counts per gene were averaged across multiple experiments per tissue. Following the same steps as in previous examples, I identified the rank number 17,086 as a cutoff point (Fig. 1F). To test if proteins shortlisted using my approach reflect the essential physiology of the heart, I submitted the list of top 209 genes, determined by the method and ranking 17,087 through 17,294 to Metascape. Top enriched biological categories were highly relevant for heart physiology and function. These categories includ for example “muscle system process”, “oxidation–reduction process” “actin filament-based process”, “smooth muscle contraction” and other (Fig. 1G). Thus, the cutoff used in this example captured essential molecular mechanisms that dominate heart physiology. These categories were enriched with a very high level of significance (-log10(*p*) > 10). Biological categories enriched in the shortlist of the lowest ranking proteins were non-relevant to known adult heart physiology (Supplemental Fig. 2C).

## Discussion and conclusions

In this study, I describe a simple and reproducible approach for the cutoff identification in descriptive high-throughput studies, which method is entirely based on the analysis of the shape of the curve of the data distribution. The major assumption of that approach is that a small number of variables with high values dominate the biological system and determine its major processes and functions. Thus, the described method for cutoff identification may be used following a visual inspection of the shape of the curve to confirm its biphasic nature to prioritize variables for more detailed functional analysis, in situations where other methods of dichotomization of data are inaccessible. As such the method should be used with a complete list of variables without prior application of other cutoff approaches.

Three different datasets analyzed here as examples demonstrate that the described cutoff identification method produces shortlists of variables highly relevant for dominant functions/pathways of the analyzed biological systems. The shortlist of highly expressed genes in the human cerebral cortex was highly enriched for categories related to synaptic transmission, nervous system development, and even higher functions, such as learning and memory. The shortlist of genes sensitive to chemical exposures was enriched for biological categories involved in response to stress and damage. Finally, the shortlist of proteins expressed highly in the human heart was significantly enriched for biological categories relevant to muscle architecture, contractions, and contraction regulation.

I should note here, that some applications may require more or less stringent criteria for the cutoff. In these situations, the described approach may still be useful as it allows to identify the point where the curve of values distribution changes most rapidly. Using this reproducibly identifiable point one may further select criteria with different percent of stringency relative to it. In other words, the cutoff point identified as described here may provide some meaningful reference value. Similarly using of *p*-value and fold change as cutoff points in omics studies are selected arbitrarily by researchers, but they represent meaningful indicators of the data structure and provide reproducibility of the data analysis.

The results of the use of the described dichotomization approach should be interpreted cautiously. For example, the fact that some gene was found in the short-list of highly expressed genes in a tissue does not necessarily mean that this gene is highly tissue-specific. In fact, many “housekeeping” genes are highly expressed in the majority of cell types [15], as they are major players of biological processes common to different cells and tissues. It is also likely that some genes with a normally low level of expression may still be important players of highly tissue-specific processes. Overall, dichotomization of continuous variables should be done with caution, as it is always associated with the cost of losing some important information [16]. Thus, in each specific situation of the use of the suggested dichotomization approach a biological relevance of the approach should be taken into consideration.

## Supplementary Information

**Additional file 1:** **Supplemental Fig. 1.** Example of normalized geneexpression values distribution based on human cerebral cortex gene expressiondata (see Example 1 in Examples of the Method Use). **Supplemental Fig. 2.** Biological categoriesenriched in shortlists of lowest-ranking variables for the following datasets:genes expressed in human cerebral cortex (A), genes sensitive to chemicalexposures (B), and proteins expressed in the adult human heart (C).

## Data Availability

The datasets used and/or analyzed during the current study are available from the following repositories: The Human Protein Atlas (9)—https://www.proteinatlas.org/; Mendeley Data (14),—https://data.mendeley.com/datasets/65fcympd2j/1; and. The Human Proteome Map (15)—http://www.humanproteomemap.org/. All data are publicly accessible.
