# Supplementary material for: Simple method for cutoff point identification in descriptive high-throughput biological studies
Source: BMC Genomics. 2022 Mar 14;23:204. doi: 10.1186/s12864-022-08427-6 (PMC8922865; doi:10.1186/s12864-022-08427-6)
Supplement: Supplementary file 1 — Additional file 1: Supplemental Fig. 1. Example of normalized geneexpression values distribution based on human cerebral cortex gene expressiondata (see Example 1 in Examples of the Method Use). Supplemental Fig. 2. Biological categoriesenriched in shortlists of lowest-ranking variables for the following datasets:genes expressed in human cerebral cortex (A), genes sensitive to chemicalexposures (B), and proteins expressed in the adult human heart (C). [file 12864_2022_8427_MOESM1_ESM.docx]

Supplemental Figure 1.


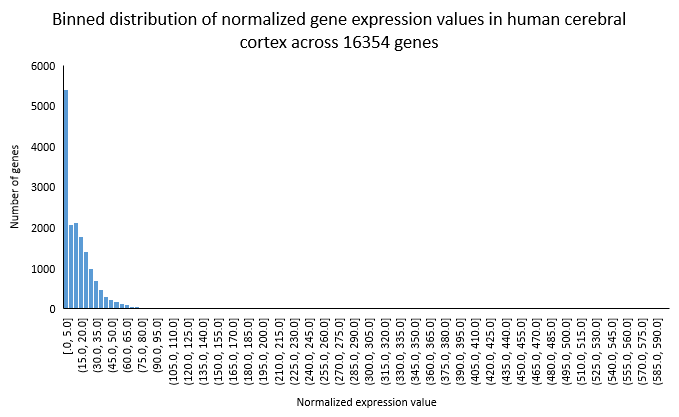


Supplemental Fig. 1. Example of normalized gene expression values distribution based on human cerebral cortex gene expression data (see Example 1 in Examples of the Method Use).


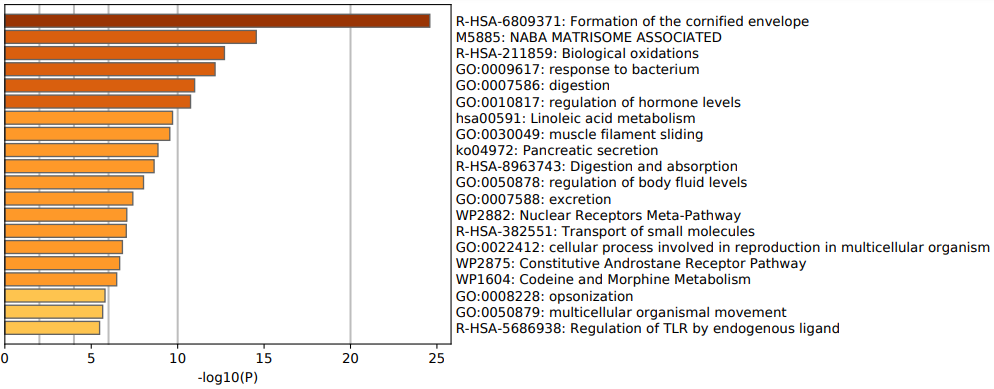
Supplemental Figure 2

A


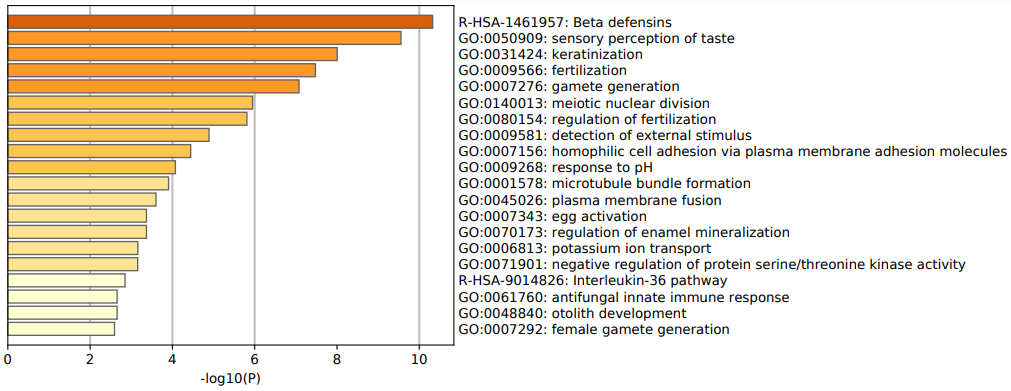


B


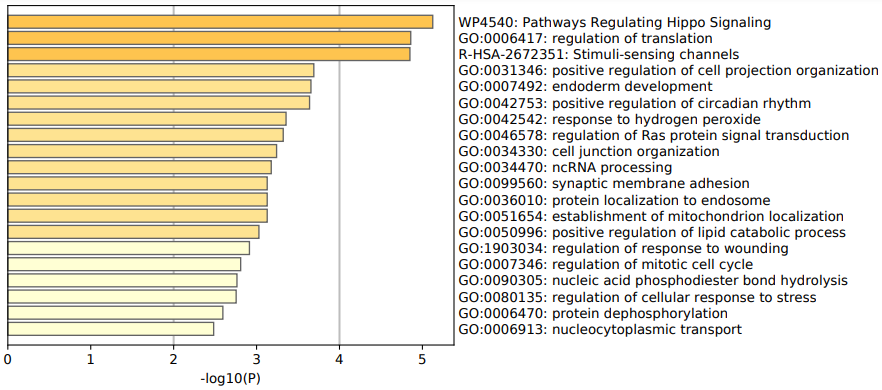


C

Supplemental Fig. 2. Biological categories enriched in shortlists of lowest-ranking variables for the following datasets: genes expressed in human cerebral cortex (A), genes sensitive to chemical exposures (B), and proteins expressed in the adult human heart (C).
